# Prevalence of SARS-Cov-2 antibodies and living conditions: the French national random population-based EPICOV cohort

**DOI:** 10.1186/s12879-021-06973-0

**Published:** 2022-01-09

**Authors:** Josiane Warszawski, Anne-Lise Beaumont, Rémonie Seng, Xavier de Lamballerie, Delphine Rahib, Nathalie Lydié, Rémy Slama, Sylvain Durrleman, Philippe Raynaud, Patrick Sillard, François Beck, Laurence Meyer, Nathalie Bajos, The EPICOV study group, Josiane Warszawski, Nathalie Bajos, Muriel Barlet, François Beck, Emilie Counil, Florence Jusot, Aude Leduc, Nathalie Lydié, Claude Martin, Laurence Meyer, Philippe Raynaud, Alexandra Rouquette, Ariane Pailhé, Nicolas Paliod, Delphine Rahib, Patrick Sillard, Alexis Spire

**Affiliations:** 1 INSERM CESP U1018, Université Paris-Saclay, AP-HP Epidemiology and Public Health Service, Service, Hôpitaux Universitaires Paris-Saclay, 82 rue du Général Leclerc, 94276 Le Kremlin-Bicêtre, France; 2 AP-HP Epidemiology and Public Health Service, Hôpitaux Universitaires Paris-Saclay, Le Kremlin-Bicêtre, France; 3 Unité des Virus Emergents, UVE, Aix Marseille Univ, INSERM 1207, IRD 190, Marseille, France; 4 Santé Publique France, Saint-Maurice, France; 5 Inserm, CNRS, Team of Environmental Epidemiology applied to Reproduction and Respiratory Health, Institute for Advanced Biosciences, University Grenoble Alpes, Grenoble, France; 6 Institut thématique de Santé Publique, INSERM, Paris, France; 7 DREES - Direction de la Recherche, des Etudes, de l’évaluation et des statistiques, Paris, France; 8 Institut National de la statistique et des études économiques, Montrouge, France; 9 IRIS, INSERM, EHESS, CNRS, Aubervilliers, France

**Keywords:** COVID-19, SARS-COV-2, Seroprevalence, Population-based survey, Random sample, Risk factors

## Abstract

**Background:**

We aimed to estimate the seroprevalence of SARS-CoV-2 infection in France and to identify the populations most exposed during the first epidemic wave.

**Methods:**

Random selection of individuals aged 15 years or over, from the national tax register (96% coverage). Socio-economic data, migration history, and living conditions were collected via self-computer-assisted-web or computer-assisted-telephone interviews. Home self-sampling was performed for a random subsample, to detect IgG antibodies against spike protein (Euroimmun), and neutralizing antibodies with in-house assays, in dried blood spots (DBS).

**Results:**

The questionnaire was completed by 134,391 participants from May 2nd to June 2st, 2020, including 17,441 eligible for DBS 12,114 of whom were tested. ELISA-S seroprevalence was 4.5% [95% CI 3.9–5.0] overall, reaching up to 10% in the two most affected areas. High-density residences, larger household size, having reported a suspected COVID-19 case in the household, working in healthcare, being of intermediate age and non-daily tobacco smoking were independently associated with seropositivity, whereas living with children or adolescents did not remain associated after adjustment for household size. Adjustment for both residential density and household size accounted for much of the higher seroprevalence in immigrants born outside Europe, twice that in French natives in univariate analysis.

**Conclusion:**

The EPICOV cohort is one of the largest national representative population-based seroprevalence surveys for COVID-19. It shows the major role of contextual living conditions in the initial spread of COVID-19 in France, during which the availability of masks and virological tests was limited.

**Supplementary Information:**

The online version contains supplementary material available at 10.1186/s12879-021-06973-0.

## Introduction

The COVID-19 pandemic has highlighted the paramount importance of public health surveys including assessments of seroprevalence for estimating the cumulative incidence of SARS-CoV-2 infection at population level. Evaluations limited to data for confirmed cases or deaths greatly underestimate disease propagation, due to the large proportion of mildly affected or asymptomatic individuals and the lack of RT-PCR screening tests at the start of the pandemic [1]. Nationwide-representative population antibody studies have been conducted in few countries to assess SARS-CoV-2 circulation, but rarely on random sample from general population [2].

France has been severely affected by COVID-19, but disease burden has been uneven across the country. Concerns about the contributions of social inequalities to spatial variations of COVID-19 exposure or severity have been raised [3], but most of the available data are based on deaths, hospitalization or reported cases [4].

EpiCOV is a large French national random population-based public health study including serological testing and longitudinal follow-up, aiming at both analysing the impact of living conditions on the dynamics of the epidemic, and the impact of the epidemic on health and living conditions[5].

Here, we aimed to provide a national estimate of SARS-Cov2 seroprevalence in France in May 2020, at the end of the first lockdown, and to identify the most exposed populations in terms of living and socio-economic conditions.

## Methods

### Study design

Individuals aged 15 years or older living in mainland France or three of the five French overseas territories were randomly selected from the FIDELI administrative sampling frame. FIDELI covers 96.4% of the population living in France, providing postal addresses for all individuals, and an e-mail address or telephone number for 83%.

Sampling was stratified for two criteria: administrative area (départements—equivalent to counties—in mainland France and three overseas territories), and a binary indicator of poverty defined on the basis of a threshold of 60% of the median national per capita household income. A differential sampling fraction was used to ensure overrepresentation of the less densely populated départements and people with lower incomes, for which lower response rates were expected. Individuals living in residential care homes for the elderly were excluded.

### Multimodal data collection

All selected individuals were contacted by post, e-mail and text messages (SMS), with up to seven reminders. Self-computer-assisted-web (CAWI) or computer-assisted-telephone interviews (CATI) was offered to a random subsample of 20%. The remaining 80% were assigned to CAWI exclusively.

### Home blood self-sampling and serological testing

Home capillary blood self-sampling was proposed during the web/telephone questionnaire. Dried-blood spots were collected on 903Whatman paper (DBS) kits set to each participant agreeing to blood sampling mailed to the central biobank (Robert Pellegrin Hospital, Bordeaux) to be punched with a PantheraTM machine (Perkin Elmer). Eluates were processed in the virology laboratory (Unité des virus Emergents, Marseille) with a commercial ELISA kit (Euroimmun®, Lübeck, Germany) for detecting anti-SARS-CoV-2 antibodies (IgG) against the S1 domain of the viral spike protein (ELISA-S), according to the manufacturer’s instructions. All samples with an ELISA-S test optical density ratio ≥ 0.7 were also tested with an in-house microneutralization assay to detect neutralizing anti-SARS-CoV-2 antibodies. For this assay, VeroE6 cells cultured in 96-well microplates, 100 TCID_50_ of the SARS-CoV-2 strain BavPat1 (courtesy of Prof. Drosten, Berlin, Germany) and serial dilutions of serum (1/20–1/160) were used, as described elsewhere [6]. Dilutions associated with the presence or absence of a cytopathic effect on 4.5 days after infection were considered negative and positive, respectively. The virus neutralization titer (VNT) referred to the highest dilution of serum with a positive result. Specimens with a VNT ≥ 40 were considered positive, as the specificity at this threshold was 100% on 486 samples collect before the emergence of SARS-Soc-2 in 2017.

For the first round of the study in May 2020, due to the logistic complexity of such rapid implementation, a national mainland subsample and six department subsamples were randomly selected for testing, including those with the highest COVID-19 prevalences at the time.

### Outcome

Seroprevalence was estimated as the proportion of the individuals tested with an ELISA-S ratio ≥ 1.1 (ELISA S +), according to the ratio threshold supplied by the manufacturer, considered as the main criteria. We also considered the proportion of individuals with neutralizing antibodies with titres ≥ 40 (SN+). Two more sensitive estimates of seroprevalence were provided: the proportion of individuals with an ELISA-S ratio ≥ 0.7, the threshold for the microneutralization assay, and the proportion of individuals with an ELISA-S+ or SN+ result.

### Exposure

We considered the contextual variables, living conditions, and individual characteristics.

As contextual variables, we considered the quintile of hospitalisation for COVID-19 and the sextile of COVID-19 death rate cumulated until the first week of May at department level, the population density in municipality of residence, and whether the neighbourhood was considered socially deprived, in accordance with national definitions for prioritising targeted socio-economic interventions.

Living conditions included the number and age of the people living in the household, overcrowding (defined as at least two people living in less than 18 m^2^ per person), and whether one of the other members of the household was reported to have had fever, cough or a positive virological test since January 2020 (suspected COVID-19 case).

The individual characteristics recorded included gender, age, tobacco use, the decile income of the household per capita, diplomas, occupation and migration history.

### Ethics and reglementary issues

This study was performed in accordance with the relevant guidelines and regulations. The survey was approved by the CNIL (the French data protection authority) (ref: MLD/MFI/AR205138) and the ethics committee (Comité de Protection des Personnes Sud Meediterranee III 2020-A01191-38) on April 2020. The survey was also approved by the “Comité du Label de la Statistique Publique”. All participants or their legally authorized representatives had provided informed consent to participation in this study. The serological results were sent to the participants by post with information about interpreting individual test results.

### Statistical analysis

SARS-Cov-2 seroprevalence was estimated with 95% confidence intervals at the national level and by geographic area, contextual variables, housing conditions, and individual characteristics. Multivariate logistic regression models included non-collinear variables identified as potential risk factors, and variables with p-values < 0.20 in univariate analysis. Univariate and multivariate analyses were conducted with ELISA-S+ as the main outcome. We considered the subpopulation of individuals not living alone for investigating the effects of the number of people living in the household, the presence of a minor (under 18 years of age) and a suspected COVID-19 case among household members.

#### Non-response adjustment weights

Final calibrated weights were calculated to correct for non-response, as detailed elsewhere [5]. The sampling weight (the inverse of inclusion probability) was first divided by the probability of response estimated with logit models adjusted for auxiliary variables potentially linked to both the response mechanism and the main variables of interest in the EpiCov survey. The Fideli sampling frame provided a wide range of auxiliary variables, including the socio-demographic variables, income distribution classes, quality of contact information, and contextual variables, such as population density, the proportion of people aged over 65 years or below the poverty line in the area, obtained by georeferencing information. Response homogeneity groups were then derived from this estimated probability (established within each department for correction for non-response to the common short questionnaire). The response probability was then estimated from the percentage of respondents in each homogeneity group, yielding first-step weights.

In the second step, these weights were calibrated according to the margins of the population census data and population projections for several variables (10-year age categories, sex, département, diploma level, and region). Weights for the serological subsample were calibrated at national and local level for the six overrepresented areas. This calculation was designed to decrease the variance and the residual bias for variables correlated with margins.

The sampling design was taken into account for estimating prevalence, and confidence intervals in statistical tests, and crude and adjusted odds ratio in logistic regression models.

Analyses were performed with SAS proc survey and STATA svy procedures.

## Results

**Fig. 1:**
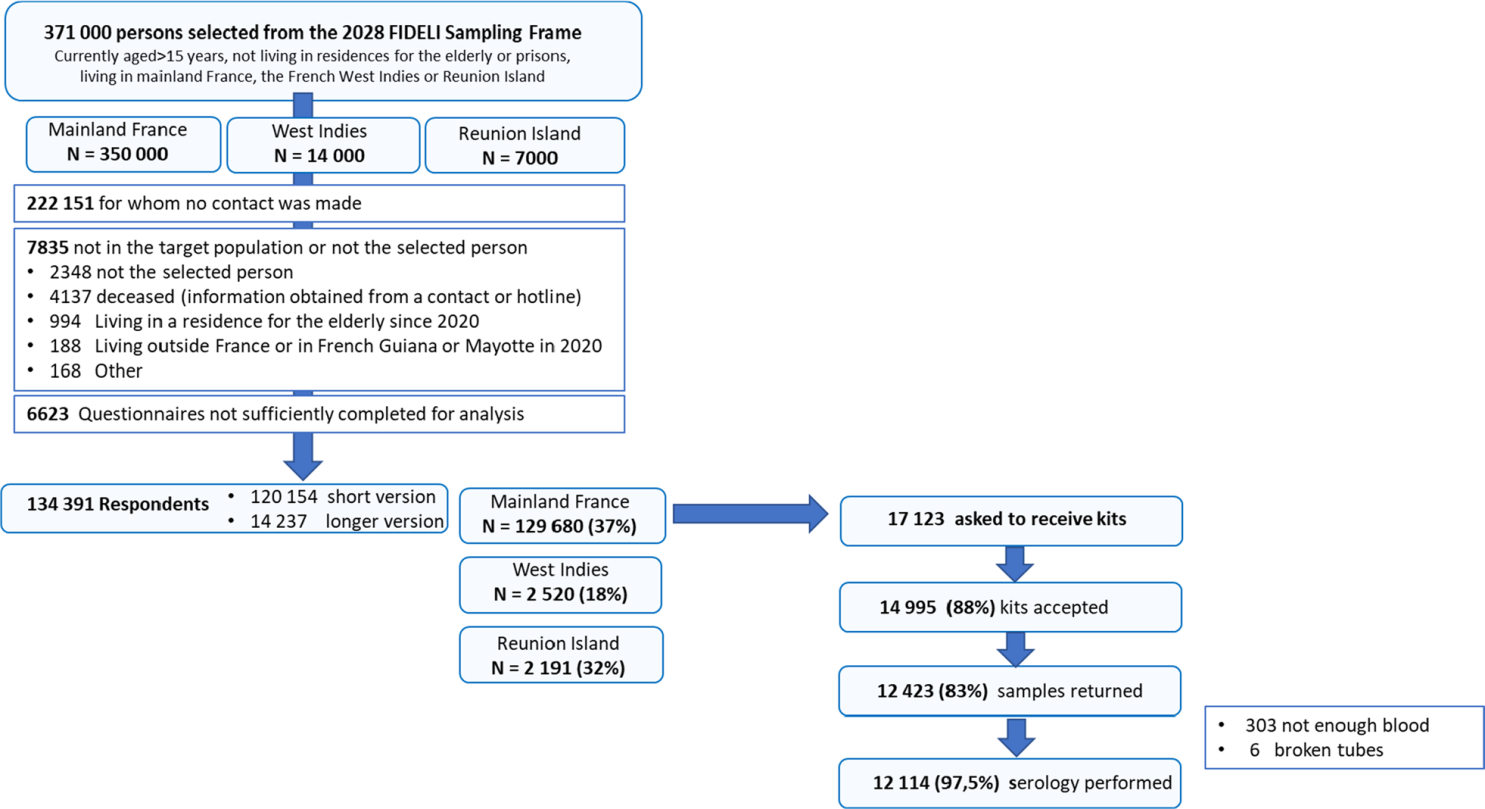
Flowchart: the national EpiCov cohort, round 1—May 2020

We selected 371,000 people aged 15 years or over at random, 134,391 of whom completed the questionnaire from May 2th to June 2th 2020. Within the random subsample of 17,123 people living in mainland France eligible for home testing, 14,995 agreed to receive the kit, 12,423 sent the DBS sample to the biobank and 12,114 samples could be analyzed (Fig. 1). The median date for blood sampling was May 21st 2020 (IQR 18th–28th May).

### National and territorial seroprevalence (Table 1, Fig. 2, Additional file 1: Table S1)

**Table 1:** Prevalence of antibodies against SARS-CoV-2^1^ in people living in France^2^ at the end of the first lockdown according to cumulative hospitalisation and death rates cumulated until the first week of May at départment level: the national EpiCov cohort, round 1—May 2020

	Total	ELISA-S+ ELISA-S ≥ 1.1			SN+ Neutralisation assay ≥ 40			ELISA-S+ or SN+			ELISA-S+/i ELISA-S ≥ 0.7		
	N	N	%^3^	95% CI^3^	N	%^3^	95% CI^3^	N	%^3^	95% CI^3^	N	%^3^	95% CI^3^
Mainland France	**12,114**	**785**	**4.5**	**[3.9–5.0]**	**656**	**4.1**	**[3.6–4.7]**	**892**	**5.5**	**[4.8–6.1]**	**1132**	**7.1**	**[6.4–7.8]**
Quintile of hospitalisation rate													
1st quintile (lowest rate)	1017	30	2.7	[1.5–3.9]	24	1.9	[1.1–2.7]	38	3.3	[2.0–4.6]	61	5.7	[4.0–7.5]
2nd quintile	1228	43	2.9	[1.9–3.8]	37	2.4	[1.5–3.2]	60	3.9	[2.8–5.0]	77	5.1	[3.8–6.3]
3rd quintile	1170	52	3.6	[2.5–4.7]	50	3.6	[2.5–4.8]	62	4.4	[3.1–5.6]	72	5.2	[3.9–6.6]
4st quintile	3378	148	4.1	[2.9–5.3]	115	4.7	[3.2–6.2]	170	5.6	[4.1–7.2]	245	7.2	[5.5–8.9]
5st quintile (highest rate)	5321	512	9.2	[7.4–10.9]	430	8.0	[6.3–9.7]	562	10.0	[8.2–11.7]	677	12.4	[10.5–14.3]
Sextile of death rate													
1st sextile (lowest rate)	734	19	2.3	[1.1–3.4]	16	1.6	[0.8–2.5]	27	3.1	[1.8–4.4]	40	4.8	[3.1–6.5]
2nd sextile	1156	26	2.7	[1.6–3.8]	31	2.3	[1.4–3.1]	47	3.6	[2.4–4.8]	67	5.4	[3.8–6.9]
3rd sextile	892	38	3.6	[2.3–4.9]	35	3.2	[2.0–4.4]	49	4.4	[3.0–5.8]	62	5.9	[4.2–7.5]
4st sextile	2393	99	3.4	[2.5–4.4]	71	3.8	[2.5–5.1]	113	4.7	[3.3–6.1]	165	5.8	[4.3–7.3]
5st sextile	1780	91	5.3	[3.5–7.1]	84	5.7	[3.7–7.6]	106	6.4	[4.4–8.5]	134	7.7	[5.5–9.9]
6st sextile (highest rate)	5159	502	9.5	[7.6–11.3]	419	8.1	[6.3–9.9]	550	10.3	[8.4–12.2]	664	12.9	[10.9–15.0]

Bold is used to underline % and OR

^1^Home sampling for finger prick/Euroimmun ELISA-S and seroneutralization tests

^2^People aged 15 or over, residing in mainland France, but not in care homes for the elderly or prisons

^3^The sampling design is taken into account for the estimation of prevalence, confidence intervals, with the SAS procsurvey procedure. The percentages are weighted by sampling weight (the inverse of inclusion probability), corrected for non-response probability and calibrated on the margin of the census. The prevalences are not equal to n/N

**Fig. 2:**
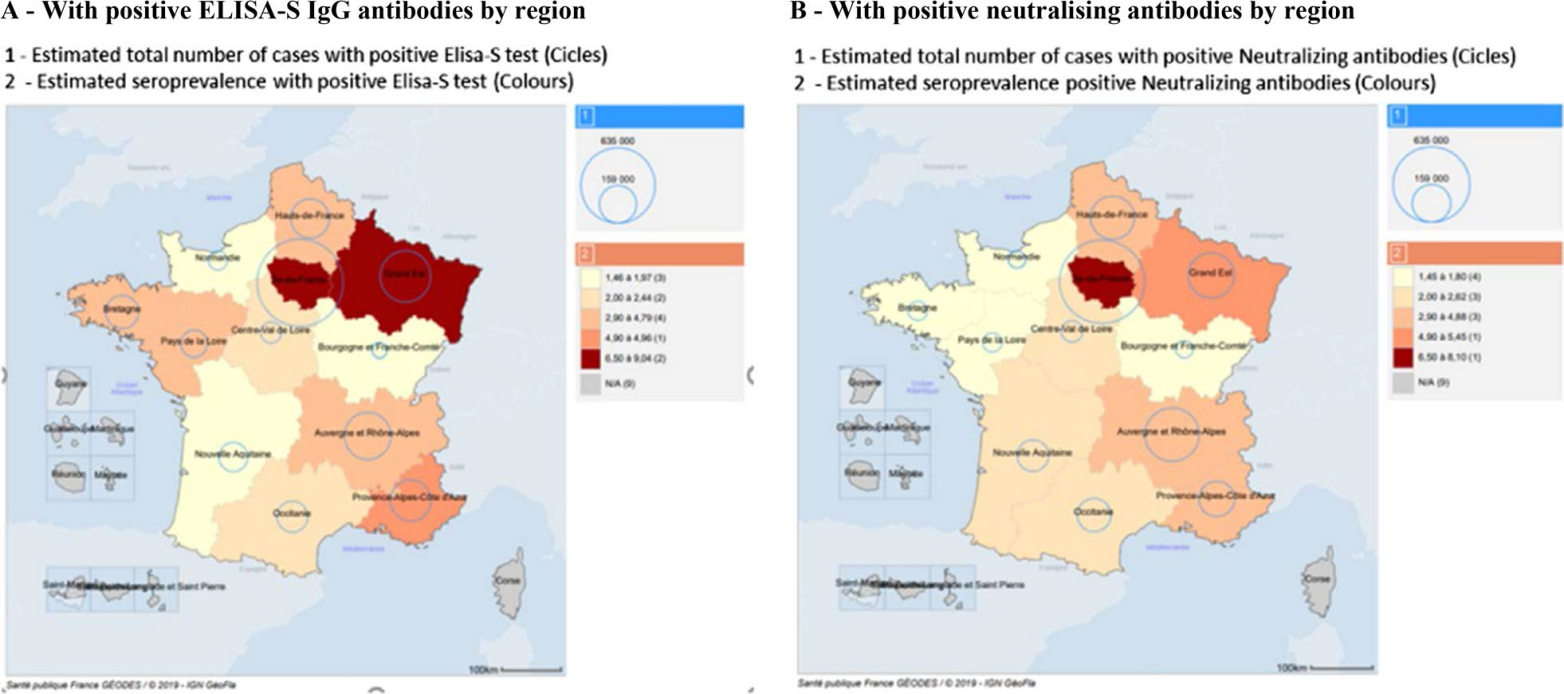
Geographic prevalence of antibodies against SARS-CoV-2^1^ in people living in France^2^ at the end of the first lockdown: the national EpiCov cohort, round 1—May 2020

For the main outcome (ELISA-S ratio ≥ 1.1), seroprevalence was 4.5% [95%CI 3.9–5.0] nationally (Table 1). Neutralizing antibodies (SN+) were detected in 4.1% [3.6–4.7] corresponding to 70.7% [65.0–76.4] of those with an ELISA-S ratio ≥ 1.1 (549/785) and 36.6% [27.7–45.4] of those with an ELISA-S ratio between 0.7 and 1.1 (107/347). Seroprevalence was 5.5% [4.8–6.1] considering all ELISA S+ or SN+ individuals, and 7.1% [6.4–7.8] if an ELISA threshold of 0.7 was used instead of 1.1. Median and inter-quartile range of Elisa-S ratio and the distribution of Virus Neutralization Titer are reported in Additional file 1: Tables S5a and b.

Considerable geographic differences were observed. In the départements with the highest and lowest cumulative death rates until May, seroprevalence was 9.5% [7.6–11.3] and 2.3% [1.1–3.4] for an ELISA-S ratio ≥ 1.1, respectively.

### Relationships between contextual living conditions and ELISA-S+ seropositivity (Tables 2 and 3)

**Table 2:** SARS-Cov-2 SEROPREVALENCE (ELISA-S ≥ 1.1^1^) according to living conditions, and individual socio-economic factors, in people living in France^2^: the national EpiCov cohort, round 1—May 2020

	N	n	%^3^	95% CI^3^	P
Population density in municipality of residence					
Low	3666	219	**3.4**	[2.6–4.3]	< 0.001
Medium	3562	199	**3.3**	[2.4–4.1]	
High	4886	367	**6.4**	[5.3–7.5]	
Living in a socially deprived neighbourhood					
No	11,589	743	**4.2**	[3.7–4.8]	0.021
Yes	525	42	**8.2**	[3.7–12.7]	
Overcrowded housing^4^					
Living alone	1665	74	**2.1**	[1.3–2.9]	< 0.001
Housing not particularly crowded	9095	588	**4.3**	[3.7–4.9]	
Crowded housing	1097	100	**9.2**	[6.1–12.4]	
Number of people in the household					
1	1665	74	**2.1**	[1.3–2.9]	< 0.001
2	4266	203	**2.7**	[2.1–3.3]	
3	2268	173	**5.2**	[3.8–6.4]	
4	2560	210	**7.1**	[5.4–8.7]	
5 or more	1349	125	**8.5**	[5.7–11.3]	
Suspected COVID cases in the household^5^					
Living alone	1665	74	**2.1**	[1.3–2.9]	< 0.001
No reported cases	8822	433	**4.0**	[3.3–4.7]	
At least one reported case	1621	278	**12.9**	[10.4–15.3]	
Minor living in the household					
Living alone	1665	74	**2.1**	[1.2–2.9]	< 0.001
No minor	6284	344	**3.8**	[3.1–4.5]	
At least one minor	4159	367	**6.9**	[5.6–8.2]	
Left usual dwelling during lockdown^6^					
No	11,414	731	**4.4**	[3.8–4.9]	0.17
Yes	700	54	**6.6**	[2.9–10.2]	
Gender					
Men	5469	321	**3.9**	[3.1–4.7]	0.053
Women	6645	464	**5.0**	[4.3–5.8]	
Age (years)					
15–20	928	51	**3.6**	[1.8–5.4]	< 0.001
21–29	1253	81	**5.7**	[3.6–7.8]	
30–49	4072	366	**6.9**	[5.8–8.1]	
50–64	3375	204	**4.5**	[3.2–5.9]	
> 64	2486	83	**1.3**	[0.9–1.8]	
Tobacco use					
Daily smoker	1995	69	**2.8**	[1.8–3.8]	0.031
Occasional smoker	470	33	**5.1**	[2.6–7.5]	
Ex-smoker	3888	253	**4.5**	[3.4–5.7]	
Non-smoker	5756	430	**5.1**	[4.2–5.9]	
Immigration status					
French native	9546	597	**4.1**	[3.5–4.7]	< 0.001
1st-generation immigrant from Europe^7^	374	24	**4.8**	[1.9–7.9]	
1st-generation immigrant from outside Europe^7^	528	55	**9.4**	[5.5–13.3]	
2nd-generation immigrant from Europe^8^	706	41	3.6	[2.0–5.3]	
2nd-generation immigrant from outside Europe	548	43	6.2	[3.4–9.0]	
Occupational status					
Healthcare profession^9^	578	74	**11.4**	[7.7–15.1]	< 0.001
Other essential profession^10^	1219	99	**5.2**	[3.6–6.9]	
Non-essential profession	4960	365	**5.7**	[4.7–6.7]	
Not occupation	5356	247	**3.0**	[2.2–3.8]	
Highest diploma attained					
< High school	4236	204	**2.8**	[2.1–3.6]	< 0.001
≥ High school and < Bachelor’s degree	4029	282	**5.8**	[4.7–6.9]	
≥ Bachelor’s degree	3849	299	**6.2**	[5.1–7.4]	
Family income per capita (deciles)					
D01 (lowest)	798	52	**5.7**	[2.5–8.9]	0.008
D02–D03	1430	86	**4.8**	[3.3–6.4]	
D04–D05	1718	97	**3.3**	[2.3–4.3]	
D06–D07	2423	128	**2.9**	[2.1–3.7]	
D08–D09	3332	237	**5.5**	[4.4–6.6]	
D10 (highest)	2112	159	**6.0**	[4.5–7.4]	
Reported testing by PCR					
Tested positive	83	74	**80.5**	[60.5–< 1]	< 0.001
Tested negative	292	22	**5.9**	[1.1–9.7]	
Result of test unknown	21	1	**0.4**	[0.4–10.1]	
Not tested	11,696	683	**4.1**	[3.6–4.7]	
Don’t know if tested	22	5	**25.3**	[0.3–50.2]	

Bold is used to underline % and OR

^1^Home sampling by finger prick/Euroimmun ELISA-S test

^2^People aged 15 years or over residing in mainland France, outside residential housing for the elderly and prisons

^3^The sampling design is taken into account for the estimation of prevalence, confidence intervals and statistical tests, with the SAS procsurvey procedure. The percentages are weighted by sampling weight (the inverse of inclusion probability), corrected for non-response probability and calibrated on the margin of the census. The prevalences are not equal to n/N

^4^Living in a housing area with less than 18 m^2^ per inhabitant

^5^Other members of the household reported by the participant as having had symptoms or positive PCR tests since February 2020

^6^First national lockdown in France: March 17th to May 11th

^7^First-generation immigrants: born non-French outside France and living permanently in France (including those who subsequently acquired French nationality)

^8^Second-generation immigrants: born and living in France, with at least one parent being a first-generation immigrant

^9^Including medical and paramedical professionals, Firefighters, Pharmacists and ambulance drivers (but not including hospital cleaners, for example)

^10^Home helps or housekeepers, food shop workers, delivery drivers, public transportation drivers, cab drivers, bank customer service or reception staff, petrol station employees, police officers, postal workers, cleaning staff, security guards, construction workers, truck drivers, farmers and social workers

**Table 3:** SARS-Cov2 SEROPREVALENCE (ELISA-S ≥ 1.1^1^) according to living conditions, and individual socio-economic factors in people living in France^2^: the national EpiCov cohort, round 1—May 2020: univariate and multivariate analysis

	Univariate analysis^3^				Multivatiate analysis^3^		
	%	ORcr	95% CI	P	OR adj	95% CI	P
Population density in municipality of usual residence							
Low	**3.4**	Ref		< 0.001	Ref		< 0.001
Medium	**3.3**	**0.9**	[0.7–1.4]		**1.1**	[0.8–1.6]	
High	**6.4**	**1.9**	[1.4–2.7]		**1.9**	[1.3–2.7]	
Number people in the household							
1	**2.1**	Ref		< 0.001	Ref		< 0.001
2	**2.7**	**1.3**	[0.8–2.1]		**1.4**	[0.8–2.3]	
3	**5.2**	**2.5**	[1.5–4.1]		**2.1**	[1.2–3.5]	
4	**7.1**	**3.6**	[2.2–5.8]		**2.5**	[1.4–4.3]	
5 or more	**8.5**	**4.4**	[2.5–7.6]		**3.5**	[1.8–6.7]	
Gender							0.13
Men	**3.9**	Ref		0.053	Ref		
Women	**5.0**	**1.3**	[1.0–1.7]		**1.2**	[0.9–1.6]	
Age (years)							
15–20	**3.6**	**0.5**	[0.3–0.8]	< 0.001	**0.5**	[0.3–1.0]	0.002
21–29	**5.7**	**0.8**	[0.5–1.2]		**0.7**	[0.5–1.1]	
30–49	**6.9**	Ref			ref		
50–64	**4.5**	**0.6**	[0.5–0.9]		**0.9**	[0.6–1.2]	
> 64	**1.3**	**0.2**	[0.1–0.3]		**0.3**	[0.2–0.6]	
Tobacco use							
Daily smoker	**2.8**	Ref		0.031	Ref		0.015
Occasional smoker	**5.1**	**1.8**	[1.0–3.5]		**2.0**	[1.0–4.0]	
Ex-smoker	**4.5**	**1.6**	[1.0–2.6]		**1.9**	[1.2–3.1]	
Non-smoker	**5.1**	**1.8**	[1.2–2.8]		**2.0**	[1.3–3.0]	
Immigration status							
French native	**4.1**	Ref		< 0.001	Ref		0.55
1st gen immigrant from Europe^4^	**4.8**	**1.2**	[0.6–2.3]		**1.4**	[0.7–2.9]	
1st gen immigrant from outside Europe^4^	**9.4**	**2.4**	[1.5–4.0]		**1.6**	[0.9–2.8]	
2nd gen immigrant from Europe^5^	3.6	**0.9**	[0.5–1.5]		**1.0**	[0.6–1.6]	
2nd gen immigrant from outside Europe^5^	6.2	**1.5**	[0.9–2.6]		**1.1**	[0.6–2.0]	
Occupational status							
Healthcare profession^6^	**11.4**	**2.1**	[1.4–3.2]	< 0.001	**2.2**	[1.4–3.3]	0.002
Other essential profession^7^	**5.2**	**0.9**	[0.6–1.3]		**1.0**	[0.7–1.5]	
Non-essential profession	**5.7**	Ref			Ref		
No occupation	**3.0**	**0.5**	[0.4–0.7]		**0.9**	[0.6–1.3]	
Highest diploma attained							
< High school	**2.8**	**0.5**	[0.3–0.7]	< 0.001	**0.7**	[0.5–0.9]	0.034
≥ High school and < Bachelor’s degree	**5.8**	Ref			Ref		
≥ Bachelor’s degree	**6.2**	**1.1**	[0.8–1.4]		**0.8**	[0.6–1.1]	
Family income per capita (deciles)							
D01 (lowest)	**5.7**	**2.0**	[1.0–3.9]	0.008	**1.6**	[0.8–3.2]	0.004
D02–D03	**4.8**	**1.7**	[1.1–2.6]		**1.7**	[1.1–2.6]	
D04–D05	**3.3**	**1.1**	[0.7–1.7]		**1.1**	[0.7–1.7]	
D06–D07	**2.9**	Ref			Ref		
D08–D09	**5.5**	**1.9**	[1.4–2.7]		**1.8**	[1.3–2.6]	
D10 (highest)	**6.0**	**2.1**	[1.5–3.1]		**1.9**	[1.3–3.0]	

Bold is used to underline % and OR

^1^Home sampling for finger prick/Euroimmun ELISA-S test

^2^People aged 15 or over, living in mainland France, but not in residential care homes for the elderly or prisons

^3^The sampling design is taken into account for the estimation of prevalence, crude and adjusted odds ratios, confidence intervals and tests, with the SAS procsurvey procedure. The percentages are weighted by sampling weight (the inverse of e inclusion probability), corrected for non-response probability and calibrated on the margin of the census. The prevalences are not equal to n/N

^4^First-generation immigrants: born non-French outside France and living permanently in France (including those who subsequently acquired French nationality)

^5^Second-generation immigrants: born and living in France, with at least one parent a first-generation immigrant

^6^Including medical and paramedical professionals, Firefighters, Pharmacists and ambulance drivers (but not including hospital cleaners, for example)

^7^Home helps or housekeepers, food shop workers, delivery drivers, public transportation drivers, cab drivers, bank customer service or reception staff, petrol station employees, police officers, postal workers, cleaning staff, security guards, construction workers, truck drivers, farmers and social workers

In the two regions most affected by the epidemic, Ile-de-France and Grand-Est, prevalence was highest in metropolitan areas. Seroprevalence (ELISA-S+) in individuals living in densely populated municipalities was twice (6.4%) that of individuals living in zones of moderate (3.4%) or low (3.3%) population density. Socially deprived neighborhoods had rates twice those of non-deprived (8.2% versus 4.2%; p = 0.019), and overcrowded housing was associated with a doubling of seroprevalence (9.2% versus 4.3%; p < 0.001).

Seroprevalence increased strongly with the number of people living in the same dwelling, from 2.1% for people living alone, to 8.5% for households with more than four members (p = 0.017). It was higher in households of more than one person including a minor (4.0% vs. 1.2%; p < 0.001). This association disappeared after adjustment for household size (Additional file 1: Table S2).

Seroprevalence was higher for participants reporting that another member of the household had presented symptoms or had a positive PCR test (12.9% versus 4.0%; p < 0.001). This association was not affected by adjustment for household size, the presence of minors or population density of the living municipality (Additional file 1: Table S2).

### Relationships between individual characteristics and ELISA-S+ seropositivity (Tables 2, 3, 4)

**Table 4:** Logistic models for studying the relationship between immigration status and seroprevalence, adjusted for contextual and individual factors, in people living in France^2^: the national EpiCov cohort, round 1—May 2020

Immigration status	Relation with serological status adjustement for: contextual factors			Relation with serological statuts adjusted for: individual factors		
	OR^3^	95% CI^3^	P-value^3^	OR^3^	95% CI^3^	P-value^3^
	Univariate			Adjusted for diploma		< 0.001
French native	Ref		** < 0.001**	Ref		** < 0.001**
1st gen immigrant from Europe^4^	**1.2**	[0.6–2.3]		**1.3**	[0.8–1.5]	
1st gen immigrant from outside Europe^4^	**2.4**	[1.5–4.0]		**2.7**	[1.7–4.4]	
2nd gen immigrant from Europe^5^	**0.9**	[0.5–1.5]		**1.0**	[0.6–1.6]	
2nd gen immigrant from outside Europe^5^	**1.5**	[0.9–2.6]		**1.6**	[0.9–2.6]	
	Adjusted for density		< 0.001	Adjusted for profession		**< 0.001**
French native	Ref		**0.078**	Ref		**0.002**
1st gen immigrant from Europe	**1.1**	[0.6–2.1]		**1.3**	[0.6–2.5]	
1st gen immigrant from outside Europe	**2.0**	[1.2–3.2]		**2.5**	[1.6–4.1]	
2nd gen immigrant from Europe	**0.9**	[0.5–1.4]		**0.9**	[0.6–1.6]	
2nd gen immigrant from outside Europe	**1.3**	[0.8–2.2]		**1.6**	[1.0–2.7]	
	Adjusted for household size		< 0.001	Adjusted for income decile		**< 0.001**
French native	**Ref**		**0.078**	Ref		**0.042**
1st gen immigrant from Europe	**1.3**	[0.7–2.5]		**1.4**	[0.7–2.7]	
1st gen immigrant from outside Europe	**2.0**	[1.2–3.2]		**2.6**	[1.5–4.5]	
2nd gen immigrant from Europe	**0.9**	[0.5–1.5]		**0.9**	[0.5–1.5]	
2nd gen immigrant from outside Europe	**1.2**	[0.7–2.1]		**1.7**	[1.0–2.8]	
	Adjusted for minor in the household		< 0.001	Adjusted for gender		0.052
French native	Ref		**0.034**	Ref		**0.037**
1st gen immigrant from Europe	**1.3**	[0.7–2.5]		**1.2**	[0.6–2.3]	
1st gen immigrant from outside Europe	**2.1**	[1.3–3.5]		**2.4**	[1.5–3.9]	
2nd gen immigrant from Europe	**0.9**	[0.5–1.5]		**0.9**	[0.5–1.5]	
2nd gen immigrant from outside Europe	**1.3**	[0.8–2.2]		**1.5**	[0.9–2.6]	
	Adjusted for overcrowded housing		< 0.001	Adjusted for age		< 0.001
French native	Ref		**0.14**	Ref		**0.024**
1st gen immigrant from Europe	**1.2**	[0.6–2.3]		**1.4**	[0.7–2.7]	
1st gen immigrant from outside Europe	**1.9**	[1.1–3.3]		**2.2**	[1.3–3.5]	
2nd gen immigrant from Europe	**0.9**	[0.5–1.5]		**1.0**	[0.6–1.6]	
2nd gen immigrant from outside Europe	**1.3**	[0.8–2.3]		**1.3**	[0.8–2.2]	
	Adjusted for deprived neighbourhood		0.21	Adjusted for tobacco use		0.024
French native	Ref		**0.064**	Ref		**0.002**
1st gen immigrant from Europe	**1.2**	[0.6–2.3]		**1.2**	[0.6–2.3]	
1st gen immigrant from outside Europe	**2.2**	[1.2–3.7]		**2.4**	[1.5–3.9]	
2nd gen immigrant from Europe	**0.9**	[0.5–1.5]		**0.9**	[0.5–1.5]	
2nd gen immigrant from outside Europe	**1.5**	[0.9–2.4]		**1.6**	[0.9–2.6]	
	Adjusted for density + household size			Adjusted for all individual factors		
French native	Ref		**0.49**	Ref		**0.0102**
1st gen immigrant from Europe	**1.2**	[0.6–2.3]		**1.6**	[0.8–3.2]	
1st gen immigrant from outside Europe	**1.5**	[0.9–2.5]		**2.4**	[1.4–4.0]	
2nd gen immigrant from Europe	**0.9**	[0.5–1.4]		**1.0**	[0.6–1.7]	
2nd gen immigrant from outside Europe	**1.0**	[0.6–1.7]		**1.5**	[0.9–2.5]	

Bold is used to underline % and OR

^1^Home sampling for finger prick/Euroimmun ELISA-S test

^2^People aged 15 or over, living in mainland France, but not in residential care homes for the elderly or prisons

^3^The sampling design is taken into account for the estimation of prevalence, crude and adjusted odds ratios, confidence intervals and tests, with the SAS procsurvey procedure. The percentages are weighted by sampling weight (the inverse of e inclusion probability), corrected for non-response probability and calibrated on the margin of the census. The prevalences are not equal to n/N. In each bivariate models, P-values are systematically given for the immigration status and for the corresponding contextual or individual adjustement variable

^4^First-generation immigrants: born non-French outside France and living permanently in France (including those who subsequently acquired French nationality)

^5^Second-generation immigrants: born and living in France, with at least one parent a first-generation immigrant

Seroprevalence tended to be higher in women than in men (5.0% versus 3.9%; p = 0.054), and increased with age, from 3.6% in people aged 15–20 to 6.9% in those aged 30–49 years, before decreasing to 1.3% in those aged 65 or over (p < 0.001). Daily smokers had a lower likelihood of having antibodies than occasional, former or non-smokers, in whom seroprevalence was similar (2.8% vs. 5%; p = 0.031).

Seroprevalence was highest in healthcare professionals (11.4%), twice that in people with other occupations self-reported as essential (5.2%) or non-essential (5.7%) during the first national lockdown (p = 0.002). Seroprevalence was 3.0%in individuals with no professional occupation.

The individuals with the lowest level of education had the lowest seroprevalence (2.8%), below those who had completed high school (5.8%) or at least a bachelor’s degree (6.2%) (p < 0.001). Concerning family income per capita, the highest seroprevalence (5% to 6%) was observed for the two lowest and the two highest deciles, with lower rates (about 3%) for central deciles (p = 0.007).

Immigration status was significantly linked to seroprevalence, which was higher in first- and second-generation immigrants born outside Europe (9.4% and 6.2%, respectively) than in non-immigrants (4.1%), or first- and second-generation immigrants from Europe (4.8 and 3.6%, respectively). The relationship between seroprevalence and immigration status from outside Europe was unaffected by adjustment for individual factors, but disappeared after adjustment for both residential population density and household size: crude ORs were 2.4 [1.5–4.0] and 1.6 [0.9–2.6] for first- and second-generation immigrants from outside Europe, whereas the adjusted ORs were 1.6 [0.9–4.0] and 1.1 [0.6–2.0], respectively (Table 4).

### Sensitivity analyses

Similar associations (Additional file 1: Tables S3, S4) were found when the analysis was restricted to individuals living in the two most affected regions (N = 5557).

Similar patterns were also observed for the proportion of individuals with SN titre ≥ 40 (Additional file 1: Table S4).

## Discussion

Epicov, designed in March 2020, just before the first national lockdown in France, aimed to estimate the proportion of the population aged 15 years or over exposed to SARS-Cov2, and to identify the subpopulations most exposed during the first epidemic wave. Overall seroprevalence was 4.5% [3.9–5.0], according to the cut-offs recommended by the manufacturer for the Euroimmun ELISA-S test that was applied on home self-sampled dried blood spots.

Only two other national serological studies based on random general population samples were performed at the same period, in Spain [7] and England [8]. They reported a prevalence of seropositivity for IgG antibodies of a similar magnitude to that in France, with a similar range of geographic disparities.

EpiCov was designed to study the effects of contextual living conditions. It showed that these conditions played a major role in the initial spread of the virus, accounting for a large proportion of exposure heterogeneity. Population density at the place of residence and household size were strongly associated with ELISA-S seropositivity, independently of individual socio-demographic and occupational characteristics. The availability of masks and tests was extremely limited until May 2020. People living in the most populous areas had little opportunity for physical distancing in current life activities outside home, particularly before lockdown.

Adjustment for both residential population density and household size accounted for much of the higher seroprevalence in immigrants outside Europe, which was twice that of the native population, whereas seroprevalence was similar in immigrants from European countries and the native population. These findings highlight the role of the spatial segregation of populations originating from low-and middle-income countries [9, 10]. Higher levels of exposure may account for part of the higher burden of COVID-19 mortality in these populations [4].

Poor socio-economic status was associated with severe COVID-19 infection [11, 12]. We found a more complex pattern for relationships with seroprevalence, which was highest in the two highest and lowest deciles of family income per capita, and lowest in the individuals with the lowest level of education. This probably reflects the combination of both high exposure to COVID-19 in qualified individuals working in care professions or having multiple social activities before lockdown, and high exposure of more deprived people living in overcrowded housing in densely populated areas, with less opportunity to telework during lockdown [13]. Seroprevalence in healthcare professionals was twice that in individuals with other occupations. Healthcare workers were highly exposed to the infection during the first wave, given the shortage of surgical masks and their proximity with patients [7, 8, 14].

Seroprevalence did not differ significantly between women and men, after adjustment for contextual and individual characteristics, including professional activity, consistent with most studies conducted in France [15, 16] and elsewhere [2, 7, 8]. Men seem to be more susceptible to develop severe forms of the infection than women [17], but there is no evidence of any difference in the probability of being infected [18].

Seroprevalence was highest at intermediate ages. Most population-based serological studies have reported a lower seroprevalence in the elderly [7, 8, 14]. Older people, at least those not living in care homes, are likely to have had fewer social interactions since being told to stay at home at the start of the outbreak. Lower rates in adolescents and young adults than in mid-age range adults have been reported in some studies [7, 19] including ours, but not in others [8, 20], and may be partly explained by school closures at the start of lockdown in France. Seropositivity was strongly associated with possible cases of infection in the same household, regardless of local population density, household size and composition. This finding is consistent with the higher risk of secondary infections among people living with others [7, 8, 21]. After adjustment for household size, seropositivity was not associated with living with a child or an adolescent under the age of 18 years. Similar results were reported in the English national seroprevalence study [8]. This finding is also consistent with smaller studies showing that the mean household secondary attack rate from adults is not significantly different from that from children, as reported in a meta-analysis [21]. By contrast, a study conducted during the same period in population cohorts in three regions of France with similar home self-sampling reported a higher seroprevalence for individuals living in households containing a young below 18 years [20]. It remains unclear whether children play a major role in intra-household transmission, which is a crucial issue, because the benefits of school closure for preventing disease spread have to be weighed up against potential psychological effects and increases in educational inequalities.

We found a strong inverse association between the presence of SARS-Cov-2 antibodies and smoking at the time of the EpiCov study, as in other studies [8, 20]. The possibility of biological mechanisms preventing infection in some smokers must be counterbalanced by evidence for higher rates of severe forms of COVID-19 in infected smokers [22].

### Strengths

The Epicov cohort is one of the largest national representative population-based surveys of seroprevalence in individuals aged 15 years and over, performed during an extremely challenging period, before summer 2020. It identified the population most affected by the initial spread of the new virus in the population, providing a basis for evaluating subsequent changes in epidemiological context and access to preventive strategies. People living below the poverty line were voluntarily over-represented in the sampling, and detailed socio-economic and migration data were available. We were therefore able to perform a powerful analysis focusing on social inequalities.

The home self-sampling with DBS detection of SARS CoV-2 antibodies limited self-selection bias, and was ideally suited to the context of the first lockdown. The acceptance of home sample was 88% and the return rate was 83%, higher than the 85% and 70% assumed for the calculation of sample size.

Non-response is a known crucial issue affecting the representativeness of population-based studies. In the EpiCov Study, a high coverage of the sampling frame, together with mixed-mode (web/telephone) data collection resulted in high quality in terms of representativeness [23]. Many auxiliary demographic and socio-economic variables were available from the sampling frame, which made it possible to correct a large part of the non-response bias. Moreover, the multimodal approach of the EpiCov provided an exceptional opportunity to correct for endogenous self-selection bias, as detailed elsewhere [5]. This bias due to the people most concerned more likely than others to participate in the study, occurs in studies dealing with topics with considerable media coverage.

### Limitations

People living in residences for the elderly were not covered by EpiCov. We cannot exclude we also missed non-dependent elderly individuals, due to hospitalization at the time of the survey, potentially contributing to the lower seroprevalence observed among the elderly.

The Euroimmun ELISA-S test has a sensitivity of 94.4%, according to the manufacturer’s cutoff. It has been evaluated in various studies, which reported a specificity ranging from 96.2 to 100% and sensitivity ranging from 86.4 to 100% [24–26]. Anti-Sars-Cov2 IgG antibody levels have been reported to decline rapidly, particularly in the elderly and in subjects with mild or asymptomatic forms [1, 27, 28]. ELISA-S IgG antibody levels may therefore have been under the manufacturer’s cut-off for some of those previously infected, With a lower threshold (0.7), seroprevalence reached 7.1% [6.4–7.8] corresponding to 3.74 million people (3.36–4.13), close to the national projections based on surveillance data [29].

EpiCov is the only national representative study to date to have reported an estimated prevalence of neutralising antibodies, at 4.1% [**3.6–4.7**]. Neutralising antibodies with a titre ≥ 40 were detected in only 70% of people ELISA-S-positive for IgG antibodies, and were also detected in 30% of participants with lower ELISA-S ratios. Several studies have reported an inverse relationship between neutralising antibody development and disease severity, but the cause-effect relationship remains unclear [30]. Neutralising antibodies may be more associated with protection against future infection, increasing survival and protection against re-infection with SARS-CoV-2 strains [31].

## Conclusion

The Epicov cohort is one of the largest national representative population-based seroprevalence surveys of individuals aged 15 years and over. It revealed a major role for contextual living conditions in the initial spread of COVID-19 in France, during a period of very limited access to prevention strategies before lockdown. It provides an exceptional tool for evaluating subsequent changes in exposure risk and, particularly, for identifying the most vulnerable populations, with changes in the epidemiological context and increases in access to testing, masks, and vaccines.

## Supplementary Information

**Additional file 1: Table S1.** Geographic prevalence of antibodies against SARS-CoV-2^1^ in people living in France^2^ at the end of the first lockdown: the national EpiCov cohort, round 1—May 2020. **Table S2.** Relationship between population density and household composition and seroprevalence ELISA-S+ (ratio ≥ 1.1^1^), in people living in France^2^ at the end of the first lockdown: national EpiCov cohort, round 1—May 2020. **Table S3.** SARS-Cov2 prevalence of neutralizing antibodies (SN ≥ 40^1^) according living conditions, and individual socio-economic factors in people living in France^2^: the national EpiCov cohort, round 1—May 2020. **Table S4.** Factors associated with detection of neutralizing antibodies (SN+  ≥ 40 IU^1^) in people living in France^2^ at the end of the first lockdown: the national EpiCov cohort, round 1—May 2020. **Table S5a.** Distribution of Elisa-S ratio and virus neutralization titer (VNT) among people with Elisa-S ratio ≥ 1.1^1^—the national EpiCov cohort^2^, round 1—May 2020. **Table S5b.** Distribution of Elisa-S ratio and virus neutralization titer (VNT) among people with Elisa-S ratio ≥ 0.7^1^—the national EpiCov cohort^2^, round 1—May 2020.

## Data Availability

The first round EpiCov dataset is available for research purpose on CASD (https://www.casd.eu/), after submission to approval of French Ethics and Regulatory Committee procedure (Comité du Secret Statistique, CESREES and CNIL).

## Abbreviations

CATI: Computer-assisted-telephone interviews
CAWI: Self-computer-assisted-web
Covid-19: Coronavirus 2019
DBS: Dried-blood spots
ELISA: Enzyme-Linked Immunosorbent Assay
ELISA S+: Positive Elisa test (ELISA-S ratio ≥ 1.1)
FIDELI: Fichiers Démographiques sur les Logements et les Individus
IgG: Immunoglobulin G
PCR test: Polymerase chain reaction
RT-PCR: Real-time reverse transcriptase-polymerase chain reaction
SN+: Positive seroneutralization test (virus neutralization titer ≥ 40)
SARS-Cov2: Severe Acute Respiratory Syndrome Coronavirus 2
TCID50: Median Tissue Culture Infectious Dose
VNT: Virus neutralization titer
