# Supplementary material for: Prevalence of SARS-Cov-2 antibodies and living conditions: the French national random population-based EPICOV cohort
Source: BMC Infect Dis. 2022 Jan 9;22:41. doi: 10.1186/s12879-021-06973-0 (PMC8743062; doi:10.1186/s12879-021-06973-0)
Supplement: Supplementary file 1 — Additional file 1: Table S1. Geographic prevalence of antibodies against SARS-CoV-21 in people living in France2 at the end of the first lockdown: the national EpiCov cohort, round 1—May 2020. Table S2. Relationship between population density and household composition and seroprevalence ELISA-S+ (ratio ≥ 1.11), in people living in France2 at the end of the first lockdown: national EpiCov cohort, round 1—May 2020. Table S3. SARS-Cov2 prevalence of neutralizing antibodies (SN ≥ 401) according living conditions, and individual socio-economic factors in people living in France2: the national EpiCov cohort, round 1—May 2020. Table S4. Factors associated with detection of neutralizing antibodies (SN+ ≥ 40 IU1) in people living in France2 at the end of the first lockdown: the national EpiCov cohort, round 1—May 2020. Table S5a. Distribution of Elisa-S ratio and virus neutralization titer (VNT) among people with Elisa-S ratio ≥ 1.11—the national EpiCov cohort2, round 1—May 2020. Table S5b. Distribution of Elisa-S ratio and virus neutralization titer (VNT) among people with Elisa-S ratio ≥ 0.71—the national EpiCov cohort2, round 1—May 2020. [file 12879_2021_6973_MOESM1_ESM.docx]

ADDITIONAL MATERIAL

Table S1: Geographic prevalence of antibodies against SARS-CoV-2^1^ in people living in France^2^ at the end of the first lockdown: the national EpiCov cohort, round 1 – May 2020

|  | Total |  | ELISA-S+  ELISA-S >1.1 | | |  | SN+  Neutralisation assay >40 | | |  | ELISA-S+ or SN+ | | |  | ELISA-S+/i  ELISA-S > 0.7 | | | | |
| --- | --- | --- | --- | --- | --- | --- | --- | --- | --- | --- | --- | --- | --- | --- | --- | --- | --- | --- | --- |
|  | N |  | N | % | 95% CI |  | N | % | 95% CI |  | N | % | 95% CI |  | N | | % | | 95% CI |
| Mainland France | 12114 |  | 785 | 4.5 | [3.9 - 5.0] |  | 656 | 4.1 | [3.6 - 4.7] |  | 892 | 5.5 | [4.8 – 6.1] |  | 1132 | 7.1 | | [6.4 – 7.8] | |
| 11- Île-de-France | 2430 |  | 214 | 9.0 | [7.1;11] |  | 172 | 8.1 | [6.2;10.0] |  | 232 | 9.8 | [7.8;11.9] |  | 290 | 11.9 | | [9.8;14.1] | |
| Paris (75) ^4^ | 1061 |  | 93 | 9.0 | [6.8;11.2] |  | 69 | 7.0 | [5.0;9.0] |  | 97 | 9.3 | [7.1;11.5] |  | 123 | 11.9 | | [9.5;14.4] | |
| Petite couronne (92. 93. 94) ^4^ | 957 |  | 93 | 9.5 | [7.1;11.8] |  | 74 | 8.1 | [5.8;10.3] |  | 103 | 10.7 | [8.2;13.3] |  | 131 | 13.4 | | [10.6;16.1] | |
| 44- Grand-Est | 3 239 |  | 323 | 6.7 | [5.1;8.3] |  | 279 | 5.5 | [4.1;6.8] |  | 357 | 7.2 | [5.5;8.8] |  | 418 | 9.5 | | [7.3;11.8] | |
| Haut-Rhin (68) ^4^ | 1 740 |  | 211 | 10.8 | [9.1;12.6] |  | 190 | 9.3 | [7.7;10.8] |  | 236 | 11.8 | [10.0;13.5] |  | 269 | 13.5 | | [11.5;15.4] | |
| Bas-Rhin (67) ^4^ | 1 191 |  | 86 | 7.6 | [5.6;9.6] |  | 66 | 5.6 | [3.9;7.2] |  | 92 | 8.1 | [6.1;10.2] |  | 114 | 9.8 | | [7.6;12] | |
| 93 -Provence-Alpes-Côte d’Azur | 1 687 |  | 69 | 5.0 | [2.8;7.1] |  | 41 | 3.6 | [1.7;5.5] |  | 78 | 5.7 | [3.5;8] |  | 125 | 7.9 | | [5.3;10.5] | |
| Bouches-du-Rhône (13) ^4^ | 1 454 |  | 56 | 3.4 | [2.3;4.4] |  | 29 | 1.9 | [1.0;2.8] |  | 61 | 3.8 | [2.6;4.9] |  | 104 | 6.1 | | [4.7;7.4] | |
| 32- Hauts-de-France | 1 499 |  | 66 | 3.7 | [1.8;5.6] |  | 59 | 4.5 | [2.2;6.8] |  | 78 | 5.5 | [3.1;7.9] |  | 102 | 6.5 | | [4;9] | |
| Oise (60) ^4^ | 1 087 |  | 49 | 3.4 | [2.4;4.4] |  | 40 | 2.7 | [1.8;3.6] |  | 54 | 3.6 | [2.6;4.7] |  | 74 | 5.3 | | [4;6.6] | |
| 84 -Auvergne-Rhône-Alpes | 716 |  | 36 | 4.0 | [2.6;5.3] |  | 35 | 4.9 | [2.9;6.8] |  | 44 | 5.7 | [3.7;7.7] |  | 53 | 6.6 | | [4.5;8.8] | |
| 76 -Occitanie | 560 |  | 19 | 2.2 | [1.1;3.4] |  | 17 | 2.6 | [1.3;4] |  | 24 | 3.2 | [1.8;4.7] |  | 33 | 4.7 | | [2.9;6.5] | |
| 75 - Nouvelle-Aquitaine | 538 |  | 13 | 2.0 | [0.8;3.1] |  | 16 | 2.1 | [1.0;3.2] |  | 20 | 2.8 | [1.5;4.1] |  | 28 | 5.0 | | [2.8;7.2] | |
| 52- Pays de la Loire | 328 |  | 11 | 2.9 | [1.1;4.7] |  | 6 | 1.5 | [0.3;2.8] |  | 15 | 4.0 | [1.9;6.1] |  | 19 | 4.9 | | [2.7;7.2] | |
| 53 - Bretagne | 307 |  | 12 | 4.8 | [1.3;8.3] |  | 6 | 1.7 | [0.3;3.2] |  | 15 | 5.4 | [1.8;9] |  | 24 | 8.2 | | [4.2;12.2] | |
| 27- Bourgogne Franche-Comté | 280 |  | 7 | 1.5 | [0.2;2.7] |  | 10 | 1.8 | [0.5;3.1] |  | 11 | 2.0 | [0.6;3.4] |  | 16 | 3.3 | | [1.4;5.2] | |
| 28- Normandie | 266 |  | 7 | 1.5 | [0.4;2.7] |  | 7 | 1.4 | [0.3;2.6] |  | 9 | 1.9 | [0.6;3.2] |  | 13 | 3.3 | | [1.4;5.1] | |
| 24 - Centre-Val de Loire | 232 |  | 8 | 2.4 | [0.6;4.2] |  | 8 | 2.5 | [0.7;4.4] |  | 9 | 2.7 | [0.8;4.6] |  | 11 | 3.5 | | [1.3;5.8] | |

Legends for Table S1

^1^Home sampling for finger prick/Euroimmun ELISA-S and seroneutralization tests

^2^ People aged 15 or over, residing in mainland France but not in residential care homes for the elderly or prisons.

^3^ The sampling design is taken into account for the estimation of prevalence, crude and adjusted odds ratios, confidence intervals and tests, with the SAS procsurvey procedure. The percentages are weighted by sampling weight (the inverse of e inclusion probability), corrected for non-response probability and calibrated on the margin of the census. The prevalences are not equal to n/N.

^4^ These départements were over-represented in the national subsample selected for COVID home sampling in this first round of the survey

Table S2 – Relationship between population density and household composition and seroprevalence ELISA-S+ (ratio > 1.1^1^), in people living in France^2^ at the end of the first lockdown: national EpiCov cohort, round 1 – May 2020

|  |  | Univariate analysis^3^ | | |  | Logistic regression 1 ^3^ | | |  | Logistic regression 2 ^3^ | | |  | Logistic regression 3 ^4^ | | |
| --- | --- | --- | --- | --- | --- | --- | --- | --- | --- | --- | --- | --- | --- | --- | --- | --- |
|  |  | OR_c_ | CI 95% | P-val |  | OR_a_ | CI 95% | P-val l |  | OR_a_ | CI 95% | P-val |  | OR_a_ | CI 95% | P-val |
| All N=12114 | Density of municipality of residence |  |  |  |  |  |  |  |  |  |  |  |  |  |  |  |
|  | Low | Ref |  | <0.001 |  |  |  |  |  |  |  |  |  | Ref |  | <0.001 |
|  | Medium | 0.9 | [0.7 - 1.4] |  |  |  |  |  |  |  |  |  |  | 1.0 | [0.7 - 1.4] |  |
|  | High | 1.9 | [1.4 - 2.6] |  |  |  |  |  |  |  |  |  |  | 1.9 | [1.4 – 2.7] |  |
|  | Household size |  |  |  |  |  |  |  |  |  |  |  |  |  |  |  |
|  | 1 | Ref |  | <0.001 |  | Ref |  |  |  | Ref |  |  |  | Ref |  | 0.001 |
|  | 2 | 1.3 | [0.8 - 2.1] |  |  | 1.3 | [0.8 – 2.1] | <0.001 |  | 1.2 | [0.7 – 1.9] | 0.001 |  | 1.2 | [0.7 – 1.9] |  |
|  | 3 | 2.5 | [1.5 - 4.1] |  |  | 2.5 | [1.5 – 4.1] |  |  | 2.0 | [1.2 – 3.4] |  |  | 2.0 | [1.2 – 3.4] |  |
|  | 4 | 3.6 | [2.2 - 5.8] |  |  | 3.4 | [1.9 – 6.2] |  |  | 2.7 | [1.4 – 4.9] |  |  | 2.8 | [1.5 – 5.1] |  |
|  | 5 or more | 4.4 | [2.5 - 7.6] |  |  | 4.2 | [2.2 – 7.8] |  |  | 3.1 | [1.6 – 6.0] |  |  | 3.1 | [1.6 – 6.0] |  |
|  | Minors ^4^ living in the household |  |  |  |  |  |  |  |  |  |  |  |  |  |  |  |
|  | None or living alone | Ref |  | <0.001 |  | Ref |  |  |  | Ref |  |  |  | Ref |  | 0.76 |
|  | Yes | 2.1 | [1.6 - 2.8] |  |  | 1.1 | [0.7 – 1.6] | 0.77 |  | 1.0 | [0.7 – 1.5] | 0.83 |  | 1.1 | [0.7 – 1.6] |  |
|  | Suspected COVID cases in the household^5^ |  |  |  |  |  |  |  |  |  |  |  |  |  |  |  |
|  | None or living alone | Ref |  | <0.001 |  | - |  |  |  | Ref |  | <0.001 |  | Ref |  | <0.001 |
|  | At least one | 3.9 | [3.0- 5.2] |  |  | - |  |  |  | 3.0 | [2.2 - 4.0] |  |  | 2.9 | [2.1 – 3.9] |  |
| Not living alone  N= 10443 | Density of municipality of residence |  |  |  |  |  |  |  |  |  |  |  |  | Ref |  | <0.001 |
|  | Low | Ref |  | <0.001 |  |  |  |  |  |  |  |  |  | 1.1 | [0.7 - 1.6] |  |
|  | Medium | 1.1 | [0.7 - 1.6] |  |  |  |  |  |  |  |  |  |  | 2.0 | [1.4 – 2.8] |  |
|  | High | 2.1 | [1.5 - 2.9] |  |  |  |  |  |  |  |  |  |  |  |  |  |
|  | Household size |  |  |  |  |  |  |  |  |  |  |  |  |  |  |  |
|  | 2 | Ref |  | <0.001 |  | Ref |  | <0.001 |  | Ref |  | 0.002 |  | Ref |  | 0.001 |
|  | 3 | 1.9 | [1.4 - 2.8] |  |  | 1.9 | [1.3 – 2.7] |  |  | 1.7 | [1.2 – 2.5] |  |  | 1.7 | [1.2 – 2.5] |  |
|  | 4 | 2.7 | [1.9 - 3.8] |  |  | 2.6 | [1.6 – 4.2] |  |  | 2.3 | [1.4 - 3.7] |  |  | 2.3 | [1.4 - 3.8] |  |
|  | 5 or more | 3.3 | [2.2 - 5.1] |  |  | 3.2 | [1.9 – 5.3] |  |  | 2.7 | [1.6 – 4.6] |  |  | 2.6 | [1.6 – 4.5] |  |
|  | Minors^4^ living in the household |  |  |  |  |  |  |  |  |  |  |  |  |  |  |  |
|  | No | Ref | [1.4 - 2.5] | <0.001 |  | Ref |  | 0.77 |  | Ref |  | 0.83 |  | Ref |  | 0.77 |
|  | Yes | 1.9 |  |  |  | 1.1 | [0.7 – 1.6] |  |  | 1.0 | [0.7 – 1.5] |  |  | 1.1 | [0.7 – 1.6] |  |
|  | Suspected COVID cases in the household^5^ |  |  |  |  | - |  |  |  |  |  |  |  |  |  |  |
|  | No | Ref |  | <0.001 |  | - |  |  |  | Ref |  | <0.01 |  | Ref |  | <0.001 |
|  | At least one | 3.6 | [2.7 - 4.7] |  |  |  |  |  |  | 3.0 | [2.2 - 4.0] |  |  | 2.9 | [2.1 – 3.9] |  |

Legends for Table S2

1. Home sampling by finger prick/Euroimmun ELISA-S test
2. People aged 15 years or over residing in mainland France, outside residential housing for the elderly and prisons.
3. The sampling design is taken into account for the estimation of prevalence, confidence intervals and statistical tests, with the SAS procsurvey procedure. The percentages are weighted by sampling weight (the inverse of inclusion probability), corrected for non-response probability and calibrated on the margin of the census. The prevalences are not equal to n/N.
4. Children or adolescent aged less than 18 years old
5. Other members of the household reported by the participant as having had symptoms or positive PCR tests since February 2020

Table S3 – SARS-Cov2 prevalence of neutralizing antibodies (SN > 40^1^) according living conditions, and individual socio-economic factors in people living in France^2^: the national EpiCov cohort, round 1 – May 2020

|  | People living in mainland France  N= 12 114 | | | | |  |
| --- | --- | --- | --- | --- | --- | --- |
|  | N | n | %^3^ | 95% CI^3^ |  |  |
| Population density in municipality of usual residence | |  |  |  |  |  |
| Low | 3666 | 188 | 2.7 | [2.0 – 3.4] | <0.001 |  |
| Medium | 3562 | 183 | 3.8 | [2.7 – 4.9] |  |  |
| High | 4886 | 285 | 5.7 | [4.5 – 6.8] |  |  |
| Number of people in the household | |  |  |  |  |  |
| 1 | 1665 | 61 | 2.4 | [1.3- 3.5] | <0.001 |  |
| 2 | 4266 | 191 | 2.8 | [2.2- 3.5] |  |  |
| 3 | 2268 | 142 | 5.1 | [3.5- 6.6] |  |  |
| 4 | 2560 | 171 | 6.2 | [4.9- 7.6] |  |  |
| 5 or more | 1349 | 91 | 6.1 | [3.5-8.7] |  |  |
| Suspected COVID cases in the household^4^ | |  |  |  |  |  |
| Living alone | 1665 | 61 | 2.4 | [1.3-3.5] | <0.001 |  |
| No reported cases | 8822 | 344 | 3.6 | [2.9-4.2] |  |  |
| At least one reported case | 1621 | 251 | 11.9 | [9.5 -14.3] |  |  |
| Gender |  |  |  |  |  |  |
| Men | 5469 | 274 | 3.8 | [2.9 - 4.6] | 0.24 |  |
| Women | 6645 | 382 | 4.5 | [3.7 - 5.2] |  |  |
| Age (years) |  |  |  |  |  |  |
| 15-20 | 928 | 42 | 3.3 | [1.5 - 5.1] | <0.001 |  |
| 21-29 | 1253 | 68 | 4.6 | [2.6 – 6.6] |  |  |
| 30-49 | 4072 | 273 | 5.8 | [4.7 – 6.8] |  |  |
| 50-64 | 3375 | 197 | 4.8 | [3.4 – 6.2] |  |  |
| > 64 | 2486 | 76 | 1.6 | [0.9 – 2.4] |  |  |
| Tobacco use |  |  |  |  |  |  |
| Daily smoker | 1995 | 57 | 2.5 | [1.5 - 3.5] | 0.042 |  |
| Occasional smoker | 470 | 27 | 5.4 | [2.3 – 6.8] |  |  |
| Ex-smoker | 3888 | 219 | 4.2 | [3.1 - 5.3] |  |  |
| Non-smoker | 5756 | 353 | 4.6 | [3.7 - 5.4] |  |  |
| Immigration status |  |  |  |  |  |  |
| French native | 9546 | 491 | 3.6 | [3.0 - 4.2] | <0.001 |  |
| 1st gen immigrant from Europe ^5^ | 374 | 22 | 3.8 | [1.5 - 6.2] |  |  |
| 1st gen immigrant from outside Europe ^6^ | 528 | 51 | 9.4 | [5.4-13.3] |  |  |
| 2nd gen immigrant from Europe ^5^ | 706 | 38 | 5.5 | [2.2 – 8.7] |  |  |
| 2nd gen immigrant from outside Europe ^6^ | 548 | 34 | 5.2 | [2.6 – 7.8] |  |  |
| Occupational status |  |  |  |  |  |  |
| Healthcare profession ^7^ | 578 | 59 | 8.9 | [5.6-12.2] | <0.001 |  |
| Other essential profession ^8^ | 1219 | 84 | 4.9 | [3.4 - 6.5] |  |  |
| Non-essential profession | 4960 | 306 | 5.0 | [4.1 – 5.9] |  |  |
| No occupation | 5356 | 207 | 3.0 | [2.2 – 3.8] |  |  |
| Highest diploma attained |  |  |  |  |  |  |
| < High school | 4236 | 195 | 3.5 | [2.5 – 4.4] | 0. 08 |  |
| ≥ High school and < Bachelor’s degree | 4029 | 225 | 4.6 | [3.6 – 5.6] |  |  |
| ≥ Bachelor’s degree | 3849 | 236 | 4.9 | [4.0 – 5.8] |  |  |
| Family income per capita (deciles) | |  |  |  |  |  |
| D01(lowest) | 798 | 53 | 5.9 | [2.7 – 9.1] | 0.17 |  |
| D02-D03 | 1430 | 70 | 4.2 | [2.7 – 5.8] |  |  |
| D04-D05 | 1718 | 77 | 3.4 | [2.1 - 4.7] |  |  |
| D06-D07 | 2423 | 113 | 3.2 | [2.3 – 4.0] |  |  |
| D08-D09 | 3332 | 193 | 4.7 | [3.6 – 5.7] |  |  |
| D10 (highest) | 2112 | 130 | 5.0 | [3.6 - 6.4] |  |  |

Legends Table S3

1. Home sampling for finger prick/seroneutralization assay
2. People aged 15 or over, living in mainland France, but not in residential care homes for the elderly or prisons.
3. The sampling design is taken into account for the estimation of prevalence, crude and adjusted odds ratios, confidence intervals and tests, with the SAS procsurvey procedure. The percentages are weighted by sampling weight (the inverse of e inclusion probability), corrected for non-response probability and calibrated on the margin of the census. The prevalences are equal to n/N.
4. Other members of the household reported by the participant as having had symptoms or positive PCR tests since February 2020
5. First-generation immigrants: born non-French outside France and living permanently in France (including those who subsequently acquired French nationality).
6. Second-generation immigrants: born and living in France, with at least one parent a first-generation immigrant
7. Including medical and paramedical professionals, Firefighters, Pharmacists and ambulance drivers (but not including hospital cleaners, for example).
8. Home helps or housekeepers, food shop workers, delivery drivers, public transportation drivers, cab drivers, bank customer service or reception staff, petrol station employees, police officers, postal workers, cleaning staff, security guards, construction workers, truck drivers, farmers and social workers.

Table S4 – Factors associated with detection of neutralizing antibodies (SN+ > 40 IU^1^) in people living in France^2^ at the end of the first lockdown: the national EpiCov cohort, round 1 – May 2020

|  | Univariate analysis^3^ | | |  | Logistic regression 2 ^3^ | | |
| --- | --- | --- | --- | --- | --- | --- | --- |
|  | OR_c_ | CI 95% | p-val |  | OR_a_ | CI 95% | p-val |
| Population density in municipality of reference |  |  |  |  |  |  |  |
| Low | Ref |  | <0.001 |  | Ref |  | 0.003 |
| Medium | 1.4 | [0.1-2.1] |  |  | 1.5 | [1.0-2.2] |  |
| High | 2.2 | [1.6-3.0] |  |  | 1.9 | [1.3-2.8] |  |
| Gender |  |  |  |  |  |  |  |
| Men | Ref |  | 0.24 |  | Ref |  | 0.54 |
| Women | 1.2 | [0.9-1.6] |  |  | 1.1 | [0.8-1.5] |  |
| Age (years) |  |  |  |  |  |  |  |
| 15-20 | 0.5 | [0.3-1.0] | <0.001 |  | 0.6 | [0.3-1.1] | 0.001 |
| 21-29 | 0.8 | [0.5-1.3] |  |  | 0.7 | [0.4-1.3] |  |
| 30-49 | ref |  |  |  | ref |  |  |
| 50-64 | 0.8 | [0.6-1.2] |  |  | 0.8 | [0.6-1.1] |  |
| > 64 | 0.3 | [0.2-0.4] |  |  | 0.3 | [0.1-0.5] |  |
| Tobacco use |  |  |  |  |  |  |  |
| Daily smoker | Ref |  | 0.037 |  | Ref |  | 0.004 |
| Occasional smoker | 2.3 | [1.1-4.7] |  |  | 2.8 | [1.3-6.1] |  |
| Ex-smoker | 1.9 | [1.2-3.0] |  |  | 2.3 | [1.4-3.8] |  |
| Non-smoker | 1.7 | [1.1-2.9 ] |  |  | 2.2 | [1.3-3.8] |  |
| Immigration status |  |  |  |  |  |  |  |
| French native | Ref |  | 0.001 |  | Ref |  | 0.15 |
| 1st generation from Europe ^4^ | 1.1 | [0.5-2.1] |  |  | 1.1 | [0.6-2.2] |  |
| 1st generation from outside Europe ^4^ | 2.8 | [1.7-4.5] |  |  | 1.9 | [1.1-3.4] |  |
| 2nd generation from from Europe ^5^ | 1.5 | [0.8-3.0] |  |  | 1.6 | [0.8-3.2] |  |
| 2nd generation from outside Europe ^5^ | 1.5 | [0.8-2.6] |  |  | 1.2 | [0.6-2.1] |  |
| Occupational status |  |  |  |  |  |  |  |
| Healthcare profession ^6^ | 1.9 | [1.2-2.9] | <0.001 |  | 1.9 | [1.2-3.1] | 0.039 |
| Essential profession ^7^ | 1.0 | [0.7-1.4] |  |  | 1.1 | [0.7-1.6] |  |
| Non-essential profession | Ref |  |  |  | Ref |  |  |
| No occupation | 0.6 | [0.4-0.8] |  |  | 0.9 | [0.6-1.5] |  |
| Highest diploma attained |  |  |  |  |  |  |  |
| < High school | Ref |  | 0.12 |  | Ref |  | 0.40 |
| ≥ High school and < Bachelor’s degree | 1.3 | [0.9-1.9] |  |  | 1.0 | [0.7-1.5] |  |
| ≥ Bachelor’s degree | 1.4 | [1.0-2.0] |  |  | 0.8 | [0.6-1.2] |  |
| Family income per capita (deciles) |  |  |  |  |  |  |  |
| D01(lowest) | 1.9 | [1.0-3.7] | 0.11 |  | 1.6 | [0.9-3.3] | 0.18 |
| D02-D03 | 1.4 | [0.9-2.2] |  |  | 1.3 | [0.8-2.0] |  |
| D04-D05 | 1.1 | [0.7-1.8] |  |  | 1.0 | [0.6-1.6] |  |
| D06-D07 | ref |  |  |  | ref |  |  |
| D08-D09 | 1.5 | [1.0-2.2] |  |  | 1.5 | [1.0-2.2] |  |
| D10 (highest) | 1.6 | [1.1-2.4] |  |  | 1.6 | [1.0-2.5] |  |

Legends Table S4

1. Home sampling for finger prick/seroneutralization assay
2. People aged 15 or over, living in mainland France, but not in residential care homes for the elderly or prisons.
3. The sampling design is taken into account for the estimation of prevalence, crude and adjusted odds ratios, confidence intervals and tests, with the SAS procsurvey procedure. The percentages are weighted by sampling weight (the inverse of e inclusion probability), corrected for non-response probability and calibrated on the margin of the census. The prevalences are equal to n/N.
4. First-generation immigrants: born non-French outside France and living permanently in France (including those who subsequently acquired French nationality).
5. Second-generation immigrants: born and living in France, with at least one parent a first-generation immigrant
6. Including medical and paramedical professionals, Firefighters, Pharmacists and ambulance drivers (but not including hospital cleaners, for example).
7. Home helps or housekeepers, food shop workers, delivery drivers, public transportation drivers, cab drivers, bank customer service or reception staff, petrol station employees, police officers, postal workers, cleaning staff, security guards, construction workers, truck drivers, farmers and social workers.

Table S5a – Distribution of Elisa-S ratio and virus neutralization titer (VNT) among people with Elisa-S ratio>1.1^1^- the national EpiCov cohort^2^, round 1 – May 2020

|  |  |  | median and IQR ELISA Ratio ^3^ | |  | % VNT^3^ | | |
| --- | --- | --- | --- | --- | --- | --- | --- | --- |
|  | N | n | Median | IQR |  | 10-20 | 40-80 | 160 |
| GLOBAL | 12114 | 785 | 2.7 | 1.7-4.6 |  | 27.6 | 22.9 | 49.5 |
| Population density in municipality of residence |  |  |  |  |  |  |  |  |
| Low | 3666 | 219 | 2.4 | 1.6-3.6 |  | 47.7 | 18.1 | 34.1 |
| Medium | 3562 | 199 | 2.7 | 1.7-3.9 |  | 23.3 | 19.5 | 57.3 |
| High | 4886 | 367 | 3.4 | 1.6-5.3 |  | 20.3 | 26.3 | 53.3 |
| Gender |  |  |  |  |  |  |  |  |
| Men | 5469 | 321 | 3.0 | 1.7-5.2 |  | 24.4 | 23.1 | 52.5 |
| Women | 6645 | 464 | 2.7 | 1.7-4.2 |  | 29.9 | 22.7 | 47.4 |
| Age (years) |  |  |  |  |  |  |  |  |
| 15-20 | 928 | 51 | 3.9 | 2.4-5.2 |  | 15.2 | 25.1 | 59.7 |
| 21-29 | 1253 | 81 | 2.7 | 1.5-3.8 |  | 36.6 | 13.6 | 49.9 |
| 30-49 | 4072 | 366 | 2.1 | 1.5-3.6 |  | 33.9 | 25.9 | 40.2 |
| 50-64 | 3375 | 204 | 4.1 | 2.3-7.5 |  | 16.4 | 22.8 | 60.7 |
| > 64 | 2486 | 83 | 3.2 | 1.8-7.6 |  | 16.8 | 20.6 | 62.6 |
| Tobacco use |  |  |  |  |  |  |  |  |
| Daily smoker | 1995 | 69 | 1.8 | 1.4-3.0 |  | 33.9 | 14.9 | 51.2 |
| Occasional smoker | 470 | 33 | 1.7 | 1.3-3.5 |  | 23.3 | 35.7 | 41.0 |
| Ex-smoker | 3888 | 253 | 2.9 | 1.8-5.2 |  | 25.6 | 19.6 | 54.8 |
| Non-smoker | 5756 | 430 | 3.1 | 1.8-4.7 |  | 27.7 | 25.2 | 47.0 |
| Immigration status |  |  |  |  |  |  |  |  |
| French native | 9546 | 597 | 2.7 | 1.7-4.0 |  | 31.1 | 20.8 | 48.1 |
| 1st generation from Europe ^4^ | 374 | 24 | 2.2 | 1.7-4.8 |  | 22.8 | 25.7 | 51.5 |
| 1st generation from outside Europe ^4^ | 528 | 55 | 4.2 | 2.7-6.4 |  | 11.1 | 36.0 | 52.9 |
| 2nd generation from from Europe ^5^ | 706 | 41 | 2.4 | 1.6-6.0 |  | 16.9 | 25.1 | 58.1 |
| 2nd generation from outside Europe ^5^ | 548 | 43 | 2.7 | 1.6-5.0 |  | 22.1 | 23.1 | 54.8 |
| Occupational status |  |  |  |  |  |  |  |  |
| Healthcare profession ^6^ | 578 | 74 | 2.4 | 1.7-4.6 |  | 25.1 | 25.0 | 49.9 |
| Essential profession ^7^ | 1219 | 99 | 2.0 | 1.5-5.0 |  | 28.7 | 22.7 | 48.6 |
| Non-essential profession | 4960 | 365 | 2.4 | 1.5-3.7 |  | 31.0 | 26.0 | 43.0 |
| No occupation | 5356 | 247 | 3.7 | 1.9-6.2 |  | 23.5 | 18.2 | 58.4 |
| Highest diploma attained |  |  |  |  |  |  |  |  |
| < High school | 4236 | 204 | 3.9 | 2.2-6.4 |  | 12.7 | 29.3 | 58.0 |
| ≥ High school and < Bachelor’s degree | 4029 | 282 | 2.4 | 1.5-4.0 |  | 30.9 | 20.2 | 48.9 |
| ≥ Bachelor’s degree | 3849 | 299 | 2.3 | 1.4-3.6 |  | 37.7 | 20.1 | 42.2 |
| Family income per capita (deciles) | |  |  |  |  |  |  |  |
| D01(lowest) | 798 | 52 | 4.2 | 2.0-6.4 |  | 15.7 | 33.3 | 51.0 |
| D02-D03 | 1430 | 86 | 2.7 | 1.7-5.0 |  | 27.1 | 19.7 | 53.2 |
| D04-D05 | 1718 | 97 | 2.5 | 1.7-5.5 |  | 35.3 | 29.8 | 34.9 |
| D06-D07 | 2423 | 128 | 2.3 | 1.6-4.0 |  | 22.7 | 22.8 | 54.5 |
| D08-D09 | 3332 | 237 | 2.4 | 1.4-3.7 |  | 27.8 | 17.3 | 54.9 |
| D10 (highest) | 2112 | 159 | 2.9 | 1.4-4.4 |  | 27.8 | 28.5 | 43.7 |

Table S5b – Distribution of Elisa-S ratio and virus neutralization titer (VNT) among people with Elisa-S ratio>0.7^1^  - the national EpiCov cohort^2^, round 1 – May 2020

|  |  | |  | | median and IQR ELISA Ratio ^3^ | |  | % VNT^3^ | | |
| --- | --- | --- | --- | --- | --- | --- | --- | --- | --- | --- |
|  | N | n | | Median | | IQR |  | 10-20 | 40-80 | 160 |
| GLOBAL | 12114 | 1132 | | 1.5 | | 0.9-3.5 |  | 40.1 | 19.5 | 40.4 |
| Population density in municipality of residence |  |  | |  | |  |  |  |  |  |
| Low | 3666 | 326 | | 1.3 | | 0.9-2.7 |  | 53.1 | 18.2 | 28.7 |
| Medium | 3562 | 294 | | 1.2 | | 0.8-2.7 |  | 37.2 | 17.7 | 45.0 |
| High | 4886 | 512 | | 1.9 | | 1.0-4.2 |  | 34.2 | 21.2 | 44.7 |
| Gender |  |  | |  | |  |  |  |  |  |
| Men | 5469 | 454 | | 1.4 | | 0.9-3.7 |  | 39.8 | 19.4 | 40.8 |
| Women | 6645 | 678 | | 1.6 | | 0.9-3.3 |  | 40.3 | 19.5 | 40.1 |
| Age (years) |  |  | |  | |  |  |  |  |  |
| 15-20 | 928 | 69 | | 3.2 | | 1.0-3.9 |  | 33.7 | 19.1 | 47.2 |
| 21-29 | 1253 | 101 | | 2.0 | | 1.1-3.2 |  | 36.5 | 12.2 | 51.4 |
| 30-49 | 4072 | 549 | | 1.4 | | 0.9-2.6 |  | 44.9 | 22.3 | 32.8 |
| 50-64 | 3375 | 276 | | 2.3 | | 0.9-4.7 |  | 28.5 | 21.3 | 50.2 |
| > 64 | 2486 | 137 | | 1.0 | | 0.8-1.9 |  | 50.9 | 13.2 | 35.9 |
| Tobacco use |  |  | |  | |  |  |  |  |  |
| Daily smoker | 1995 | 105 | | 1.3 | | 0.9-1.9 |  | 43.0 | 18.0 | 39.0 |
| Occasional smoker | 470 | 43 | | 1.5 | | 0.9-3.2 |  | 23.5 | 47.4 | 29.0 |
| Ex-smoker | 3888 | 367 | | 1.5 | | 0.9-3.5 |  | 41.9 | 17.3 | 40.8 |
| Non-smoker | 5756 | 617 | | 1.7 | | 0.9-3.7 |  | 39.7 | 19.0 | 41.3 |
| Immigration status |  |  | |  | |  |  |  |  |  |
| French native | 9546 | 865 | | 1.5 | | 0.9-3.2 |  | 41.9 | 18.7 | 39.4 |
| 1st generation from Europe ^4^ | 374 | 29 | | 1.7 | | 0.9-4.4 |  | 42.7 | 18.5 | 38.7 |
| 1st generation from outside Europe ^4^ | 528 | 69 | | 3.7 | | 1.1-5.7 |  | 23.1 | 32.4 | 44.5 |
| 2nd generation from from Europe ^5^ | 706 | 65 | | 0.9 | | 0.8-2.2 |  | 35.1 | 20.1 | 44.8 |
| 2nd generation from outside Europe ^5^ | 548 | 67 | | 1.6 | | 0.8-3.5 |  | 45.4 | 15.0 | 39.6 |
| Occupational status |  |  | |  | |  |  |  |  |  |
| Healthcare profession ^6^ | 578 | 99 | | 1.8 | | 1.2-4.0 |  | 39.5 | 19.3 | 41.1 |
| Essential profession ^7^ | 1219 | 151 | | 1.4 | | 0.9-2.2 |  | 43.5 | 21.2 | 35.3 |
| Non-essential profession | 4960 | 516 | | 1.6 | | 0.9-3.1 |  | 38.9 | 22.5 | 38.6 |
| No occupation | 5356 | 366 | | 1.4 | | 0.9-3.9 |  | 40.4 | 15.6 | 44.0 |
| Highest diploma attained |  |  | |  | |  |  |  |  |  |
| < High school | 4236 | 301 | | 1.2 | | 0.8-4.1 |  | 34.6 | 22.7 | 42.7 |
| ≥ High school and < Bachelor’s degree | 4029 | 412 | | 1.7 | | 1.0-3.2 |  | 41.8 | 17.7 | 40.5 |
| ≥ Bachelor’s degree | 3849 | 419 | | 1.5 | | 0.9-3.1 |  | 45.0 | 17.5 | 37.5 |
| Family income per capita (deciles) | |  | |  | |  |  |  |  |  |
| D01(lowest) | 798 | 71 | | 2.4 | | 0.9-4.8 |  | 22.3 | 32.3 | 45.3 |
| D02-D03 | 1430 | 122 | | 1.9 | | 1.0-3.9 |  | 37.0 | 19.7 | 43.2 |
| D04-D05 | 1718 | 150 | | 1.1 | | 0.8-2.5 |  | 46.0 | 19.4 | 34.6 |
| D06-D07 | 2423 | 197 | | 1.2 | | 0.9-2.7 |  | 40.5 | 20.1 | 39.5 |
| D08-D09 | 3332 | 329 | | 1.4 | | 0.9-3.1 |  | 43.1 | 14.0 | 42.9 |
| D10 (highest) | 2112 | 228 | | 1.5 | | 1.0-3.5 |  | 38.2 | 23.2 | 38.6 |

Legends Table S5a and S5b

1. Home sampling for finger prick /Euroimmun ELISA-S and seroneutralization tests
2. People aged 15 or over, living in mainland France, but not in residential care homes for the elderly or prisons.
3. Median, IQR and percentages are weighted by sampling weight (the inverse of e inclusion probability), corrected for non-response probability and calibrated on the margin of the census.
4. First-generation immigrants: born non-French outside France and living permanently in France (including those who subsequently acquired French nationality).
5. Second-generation immigrants: born and living in France, with at least one parent a first-generation immigrant
6. Including medical and paramedical professionals, Firefighters, Pharmacists and ambulance drivers (but not including hospital cleaners, for example).
7. Home helps or housekeepers, food shop workers, delivery drivers, public transportation drivers, cab drivers, bank customer service or reception staff, petrol station employees, police officers, postal workers, cleaning staff, security guards, construction workers, truck drivers, farmers and social workers.
